# Default-threshold operating-point validation of a commercial chest radiograph AI system for selected CT-anchored thoracic findings: a bi-national multicenter retrospective study

**DOI:** 10.1038/s41598-026-55487-9

**Published:** 2026-07-25

**Authors:** Yeliz Basar, Mustafa Ege Seker, Galina Ivanova Kirova-Nedyalkova, Elina Plamenova Milkovska, Boyan Emilov Manov, Atahan Tamturk, Ilke Tasci, Melih Karadag, Nuri Sarac, Selin Ardali Duzgun, Erencan Karakoc, Ahmet Karabulut, Cemre Kavi, Kaan Buyukkirli, Mehmet Onur Onal, Deniz Alis, Recep Savas, Ercan Karaarslan, Gamze Durhan

**Affiliations:** 1 Department Radiology, Acibadem Maslak Hospital, Istanbul, Turkey; 2 Department of Radiology, University of Wisconsin–Madison, Madison, WI USA; 3 Imaging Department, Acibadem City Clinic Tokuda Hospital, Sofia, Bulgaria; 4 Department of Radiology, School of Medicine, Hacettepe University, Ankara, Turkey; 5 Department of Radiology, School of Medicine, Ege University, Izmir, Turkey; 6 Department of Radiology, Adiyaman Training and Research Hospital, Adiyaman, Turkey; 7 Department of Radiology, Cerrahpasa Faculty of Medicine, Istanbul University–Cerrahpasa, Istanbul, Turkey; 8 Department of Radiology, School of Medicine, Acibadem Mehmet Ali Aydinlar University, Istanbul, Turkey

**Keywords:** Artificial intelligence, Chest radiography, Computed tomography, Diagnostic accuracy, Thoracic imaging, Multicenter validation, Clinical decision support, Diseases, Health care, Medical research

## Abstract

This retrospective bi-national multicenter diagnostic accuracy study evaluated the locked default-threshold performance of a commercial chest radiograph AI system for selected thoracic findings using a CT-anchored, radiographic-detectability reference framework. Consecutive eligible adult patients who underwent frontal chest radiography and chest CT at four tertiary-care centers in Turkey and Bulgaria between June and December 2024 were included. Reference labels were assigned using temporally paired CT, with adjudication of whether CT-confirmed abnormalities had a corresponding radiographic manifestation on the paired chest radiograph. For potentially dynamic findings, the allowable CT–CXR interval was restricted to ≤ 2 days. The AI system was evaluated at the manufacturer’s default threshold of 0.50. Diagnostic performance was summarized using prevalence, sensitivity, specificity, positive predictive value, negative predictive value, and false-positive burden with 95% confidence intervals. The study included 940 patients with paired CXR-CT examinations. Reference-positive prevalence ranged from 2.2% for pneumothorax to 21.0% for pleural effusion. Sensitivity was highest for fracture (91%) and pneumothorax (90%) and lowest for atelectasis (64%); specificity ranged from 82% for consolidation/opacity to 98% for fracture and pneumothorax. PPV ranged from 43% to 63%, indicating a non-trivial false-positive burden at the evaluated operating point. Because continuous probability scores, human-reader comparison, and workflow outcomes were unavailable, these findings should be interpreted as default-threshold technical validation and support further evaluation of the system as radiologist-supervised decision support with local performance monitoring, rather than standalone diagnosis.

**Supplementary Information:**

The online version contains supplementary material available at 10.1038/s41598-026-55487-9.

## Introduction

Chest radiography is one of the most widely performed diagnostic imaging procedures globally and remains a first-line modality for evaluating a broad spectrum of thoracic abnormalities, including infections, malignancies, trauma, and cardiopulmonary disease^1,2^. Despite its ubiquity and cost-effectiveness, chest X-ray (CXR) interpretation remains inherently challenging. Overlapping anatomical structures, variable technical quality, and subtle pathological signs contribute to diagnostic uncertainty and interobserver variability, particularly in high-pressure or resource-constrained settings^3,4^.

The integration of artificial intelligence (AI), particularly deep learning, into radiologic practice has shown substantial promise in supporting chest radiograph interpretation. Algorithms trained on large annotated datasets may help identify complex imaging patterns, assist radiologists in detecting clinically relevant abnormalities, support case prioritization, and improve reporting efficiency in selected settings^5^. Several studies have reported diagnostic performance comparable to expert readers for specific thoracic findings, including pneumothorax, consolidation, and pleural effusion; however, such performance is task-specific and may vary according to dataset composition, reference standard, acquisition technique, and clinical environment^6,7^. Moreover, much of the available evidence remains derived from single-center or geographically limited cohorts, often from Western academic institutions, with relatively limited assessment across diverse patient populations, imaging protocols, equipment vendors, and real-world workflows^8,9^.

Deep learning systems for chest radiography have shown promising performance in controlled validation settings, but their performance is not necessarily transportable across institutions, clinical workflows, or acquisition environments. Differences in patient case mix, disease prevalence, PA versus AP projection, portable versus fixed-unit acquisition, detector hardware, image post-processing, and local reporting practices can introduce domain shift. Prior chest radiograph AI studies have shown that model performance may decrease during external validation and that site-specific imaging or workflow features may confound disease prediction. Therefore, multicenter validation should not only report aggregate performance but also evaluate center-level variability and possible contributors to performance heterogeneity^10,11^.

Reference-standard construction is another central methodological challenge in chest radiograph AI validation. Many prior AI validation studies have used radiologist consensus based on the index radiograph as the reference standard, whereas computed tomography (CT) can improve ascertainment of several thoracic abnormalities^10–13^. However, CT is not automatically an ideal ground truth for a radiographic detection task. CT may identify abnormalities that are not visible or not reasonably reportable on CXR, which can inflate apparent false-negative rates if all CT-positive findings are treated as radiograph-detectable disease. Conversely, CT-guided review of the paired radiograph can better align the reference standard with the CXR detection task, but this creates a hybrid framework that includes reader judgment regarding radiographic visibility.

In addition, CT-paired validation cohorts may introduce verification and spectrum bias because patients who undergo CT are not representative of all patients undergoing chest radiography. Such cohorts may be enriched for clinically suspected, more severe, or more complex disease, thereby altering disease prevalence and affecting prevalence-dependent performance measures such as positive predictive value and negative predictive value. For these reasons, CT-anchored CXR AI studies should explicitly define whether they evaluate CT-defined disease, CT-confirmed CXR-visible disease, or both^14,15^.

The primary objective of this study was to evaluate the locked default-threshold operating-point performance of a commercially available MDR-CE–marked chest radiograph AI system for selected CT-confirmed thoracic findings adjudicated for radiographic visibility. Secondary objectives were to quantify abnormality prevalence, false-positive burden, and center-level performance variability as indicators of transportability across real-world clinical environments.

## Materials and methods

### Study sample

This retrospective, multicenter diagnostic accuracy study was conducted at four tertiary-care academic hospitals in two countries: Center A, Acibadem Maslak Hospital, Istanbul, Turkey; Center B, Ege University Hospital, Izmir, Turkey; Center C, Hacettepe University Hospital, Ankara, Turkey; and Center D, Acibadem City Clinic Tokuda Hospital, Sofia, Bulgaria. Institutional Review Board approval was obtained, and the requirement for informed consent was waived because of the retrospective study design (date: 21/11/2024; approval number: 2024-18/676). The study was reported in accordance with STARD guidelines^16^.

Consecutive eligible adult patients aged ≥ 18 years were identified from the subset of patients who underwent frontal chest radiography, either anteroposterior or posteroanterior projection, and chest CT within the predefined temporal window between June 2024 and December 2024.

Because eligibility required clinically performed chest CT, the cohort should be interpreted as a CT-verified clinical cohort rather than a consecutive all-comer chest radiograph population. This sampling frame may enrich the cohort for thoracic abnormalities and may not reflect the prevalence distribution of routine outpatient, screening, or emergency CXR populations. Accordingly, prevalence-dependent metrics, including positive and negative predictive values, were interpreted in relation to the study-specific abnormality prevalence.

Chest CT was used as the anchoring modality for confirming abnormalities. For potentially dynamic findings, including pleural effusion, consolidation/opacity, pneumothorax, and pulmonary edema, the maximum allowable CT–CXR interval was restricted to ≤ 2 days. For the remaining target findings, CT examinations were eligible if performed within 14 days of the corresponding chest radiograph. This abnormality-specific interval was used to reduce temporal misclassification for rapidly evolving findings while preserving the evaluation of more structural or less rapidly changing abnormalities. Nevertheless, residual temporal mismatch remained possible and was considered a limitation.

**Fig. 1:**
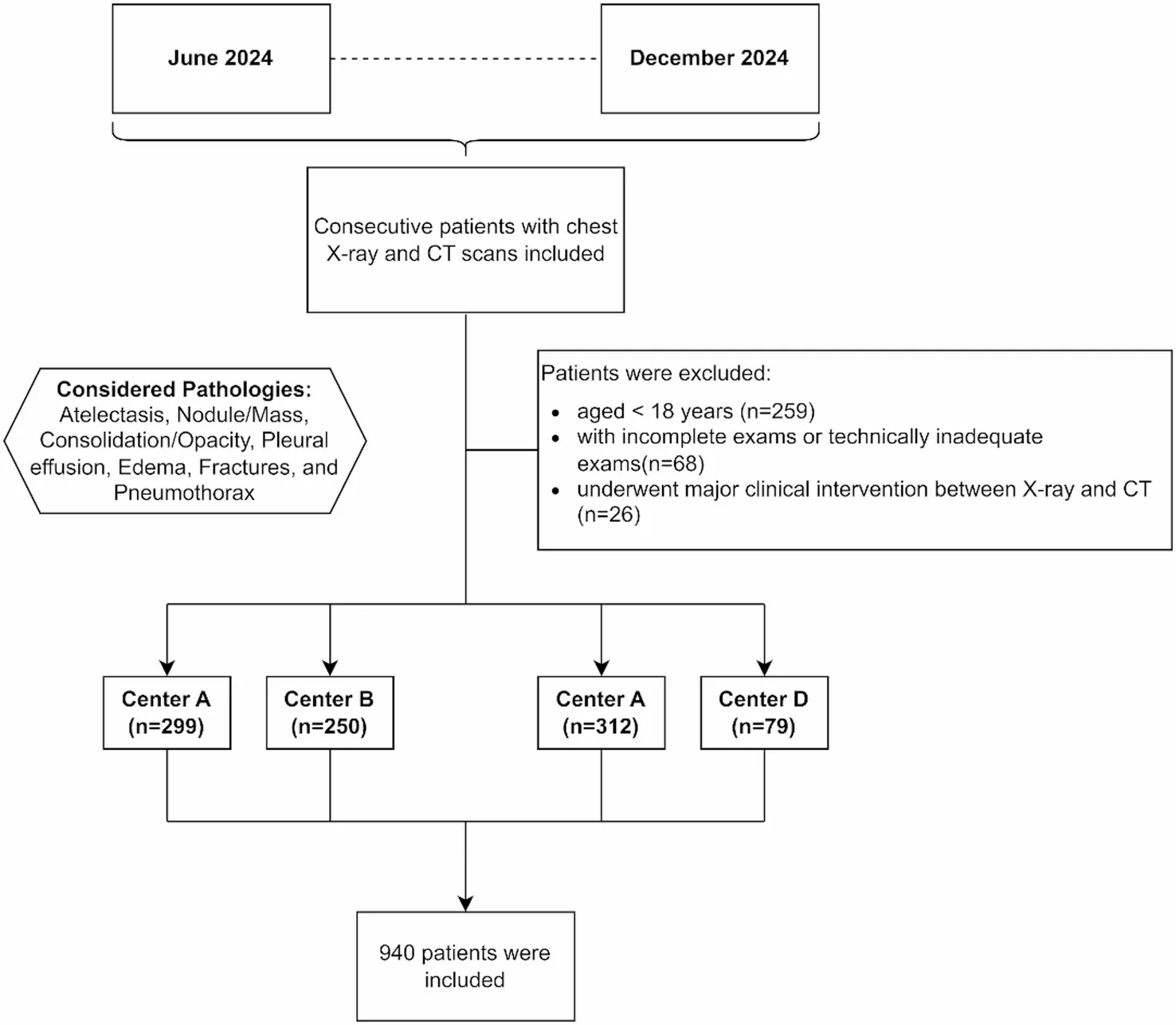
Flowchart of the study (CT: computed tomography; DL: deep learning).

Patients were excluded if either imaging modality was incomplete or technically inadequate (*n* = 68), or if a major intervening clinical event or intervention occurred between the two examinations, such as surgery or new escalation to intensive care (*n* = 26). This exclusion criterion was intended to reduce temporal misclassification between CT and CXR; however, it may have excluded some clinically unstable patients and may therefore limit generalizability. The study flowchart is shown in Fig. 1.

### Data collection

Chest radiographs were acquired using digital radiography systems. The system used for imaging at Center A was a Siemens Ysio system (Munich, Germany); at Center B were Samsung GU60A (Seoul, South Korea) and GE Definium Tempo Pro (Boston, Massachusetts) systems; at Center C were GE Definium 8000 (Boston, Massachusetts), Fujifilm FDR Smart FGXR-68 S (Tokyo, Japan) and Siemens Multifix Fusion Max (Munich, Germany) systems; and at Center D were GE Definium Tempo and Optima XR240amx (Boston, Massachusetts) systems. All examinations adhered to standard clinical protocols. Importantly, the data analyzed were not utilized in the model development.

### The AI system

The MDR-CE–marked hChestXR v1.0 (HeviAI, Istanbul, Turkey) was used in this study. According to the manufacturer user manual, hChestXR was developed using a training dataset of over 300,000 posteroanterior (PA) or anteroposterior (AP) chest radiographs acquired with mobile or fixed units. The software is designed to detect and localize seven predefined target findings: atelectasis, nodule/mass, consolidation/opacity, pleural effusion, pulmonary edema, fracture, and pneumothorax.

hChestXR analyzes each frontal chest radiograph once and generates simultaneous multi-label outputs across the seven target findings. Thus, the output for a given examination consists of seven class-specific results rather than a single mutually exclusive multi-class diagnosis or a sequential stepwise binary process. Multiple target findings may be positive in the same examination.

For each target finding, the software generates a binary output at the manufacturer’s default threshold of 0.50. Outputs meeting or exceeding this threshold were interpreted as positive. For positive outputs, the software provides one or more class-specific bounding-box localization overlays. Bounding-box overlays are available for all seven target findings and are returned to PACS as secondary-capture/PDF output together with predefined textual output. The software does not provide validated lesion-size measurements or formal lesion counts, although multiple bounding boxes may be displayed when multiple candidate abnormalities are detected.

For this retrospective analysis, binary per-finding outputs and bounding-box localization overlays generated at the manufacturer’s default threshold were available. Continuous probability scores were not available for analysis; therefore, AUROC, calibration, and threshold-sensitivity analyses could not be performed. No model retraining, fine-tuning, threshold optimization, or site-specific recalibration was performed.

### Reference standard

At each participating center, the reference standard was established by a local reader team consisting of a thoracic radiologist with at least 5 years of experience and at least one radiology resident involved in the study. The reader team reviewed the eligible chest CT examinations and their corresponding index frontal chest radiographs for reference labeling. Labels were assigned by consensus. When diagnostic uncertainty remained, a second thoracic radiologist with at least 5 years of experience was consulted, and the final label was determined by consensus.

Chest CT was used as the anchoring modality to determine the presence or absence of seven predefined target abnormalities: atelectasis, pulmonary edema, fracture, nodule, consolidation/opacity, pleural effusion, and pneumothorax. The target abnormalities were not mutually exclusive; a single examination could be reference-positive for more than one target finding. Because chest radiography is a projectional imaging technique with limited soft-tissue contrast and substantial anatomical overlap, CT-confirmed abnormalities may not always have a dependable radiographic correlate on CXR. Therefore, each CT-confirmed abnormality was subsequently adjudicated as CXR-visible or CXR-inapparent according to whether a corresponding radiographic manifestation was considered reasonably identifiable on the paired index CXR. This CT-anchored, CXR-detectability framework is consistent with prior CXR AI validation studies that distinguished CT-defined disease from CT-confirmed findings visible on chest radiographs^14,17^.

This framework was selected because CT-only reference standards may include abnormalities that are not visible or reasonably reportable on frontal chest radiography, whereas radiograph-only reference standards may miss subtle abnormalities that are better ascertained by CT. Prior CT-anchored CXR AI studies have shown that diagnostic performance estimates differ substantially depending on whether CT-defined disease or CT-confirmed CXR-visible disease is used as the reference standard^14,17^. Therefore, the present study used CT to confirm abnormality presence and paired CXR review to determine whether the abnormality constituted a reasonable radiographic detection target.

CXR-inapparent findings were defined as CT-confirmed abnormalities without a reliable radiographic correlate because of minimal disease extent, technical or anatomical limitations, superimposition, or obscuration by adjacent dominant abnormalities. Examples included nodules/masses smaller than 6 mm, linear or subsegmental atelectasis without a definite radiographic correlate, small-volume pleural effusions without visible costophrenic angle blunting or layering opacity, and abnormalities obscured by consolidation, pleural fluid, mediastinal structures, or osseous overlap. Nodules/masses smaller than 6 mm were considered CXR-inapparent because lesions of this size are often not consistently detectable on chest radiographs despite being visible on CT. This cutoff was selected to avoid overinterpreting sub-radiographic nodules/masses as radiographically detectable targets and was consistent with clinically meaningful nodule-size thresholds used in CT-based lung nodule management frameworks, including Lung-RADS^18^.

For the primary analysis, reference-positive cases were defined as CT-confirmed abnormalities adjudicated as radiographically visible on the paired CXR. CT-confirmed findings categorized as CXR-inapparent were retained in the dataset but were not considered reference-positive findings for the primary radiographic-detectability analysis. Therefore, the primary analysis evaluated AI performance for CT-confirmed abnormalities considered reasonably detectable on the index CXR, rather than for all CT-detectable disease.

Radiologists were blinded to AI outputs during CT interpretation and CXR visibility adjudication. AI outputs were reviewed only after completion of reference labeling.

For each target abnormality, AI outputs were compared with the final reference labels and categorized at the examination level as true positive, false positive, false negative, or true negative. Classification incorporated both finding class and location concordance. A positive AI output was credited as a true positive only when at least one bounding box for the same target finding corresponded to the location of a CT-confirmed, radiographically visible abnormality on the paired index CXR. If multiple same-class lesions were present, localization of at least one reference-positive target lesion was sufficient for examination-level true-positive classification for that finding.

A reference-positive examination was classified as false negative when no same-class AI bounding box corresponded to the radiographically visible reference abnormality. A positive AI output was classified as false positive when the bounding box did not correspond to a CT-confirmed, radiographically visible abnormality of the same class on the paired CXR, including cases in which the AI localized the correct abnormality class to an unrelated location. Cases in which neither the reference standard nor the AI system identified a radiographically visible target abnormality were classified as true negative.

The primary analysis was therefore finding-level and examination-level, with location-concordance adjudication, rather than a lesion-level localization analysis.

Because reference labeling and CXR visibility adjudication were performed through consensus review rather than independent double reading, interobserver agreement statistics were not calculated. The consensus approach was used to generate final reference labels for this retrospective multicenter validation; however, the reproducibility of both CT-based labeling and CXR visibility adjudication could not be quantified.

### Statistical analyses

All statistical analyses were performed using R (R Foundation for Statistical Computing, Vienna, Austria; version 4.1.0). Descriptive statistics were used to summarize patient demographics, including age (reported as mean ± standard deviation) and sex distribution. Age distribution of females and males were compared with Mann-Whitney test. The diagnostic performance of the AI system was evaluated using standard confusion matrix–based metrics and compared against the CT-confirmed reference standard.

Diagnostic performance was calculated at the examination level for each target abnormality. Although bounding-box location was considered when determining whether an AI-positive output corresponded to the reference abnormality, lesion-level sensitivity, lesion count, lesion-size estimation, and quantitative localization accuracy metrics, such as intersection-over-union, were not evaluated.

Continuous probability scores were not available in the analysis dataset. Therefore, AUROC, calibration curves, Brier score, threshold-sensitivity analyses, and decision-curve analyses were not performed. The analysis was therefore restricted to the manufacturer’s locked default operating point.

For each target pathology, the following patient-level performance metrics were calculated: accuracy, sensitivity, specificity, positive predictive value (PPV), and negative predictive value (NPV). 95% confidence intervals (95% CIs) for these proportions were computed using the Wilson score method. Between-center differences in accuracy, sensitivity, specificity, PPV, and NPV were evaluated using Pearson’s chi-squared test when expected cell counts were adequate and Fisher’s exact test when expected cell counts were small. Post-hoc pairwise comparisons between centers were performed with Bonferroni correction. Because several findings had sparse positive counts including pneumothorax, fracture, and pulmonary edema, these analyses were considered exploratory and were not interpreted as definitive evidence of site-level performance differences or causal domain-shift mechanisms.

## Results

**Fig. 2:**
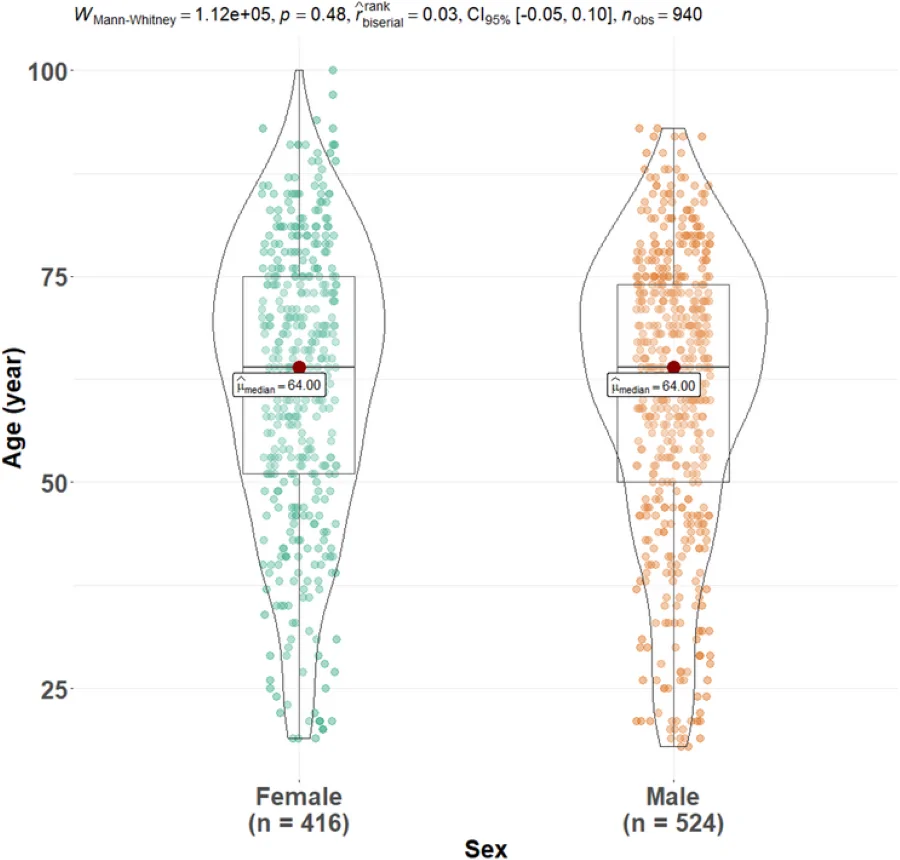
Age distribution of the patients.

The analysis included 940 adult patients with paired chest radiographs and chest CT examinations. Mean patient age was 61.4 ± 17.8 years, with a range of 18–100 years and an interquartile range of 51–74 years. Overall, 416 patients (44%) were female and 524 (56%) were male. There was no statistically significant age difference between female and male patients (*p* = 0.48). The age distribution is shown in Fig. 2. All patients had paired CXR and chest CT within the predefined temporal window; for potentially rapidly evolving abnormalities, including pleural effusion, consolidation/opacity, pneumothorax, and pulmonary edema, the CXR–CT interval was restricted to ≤ 2 days.

**Fig. 3:**
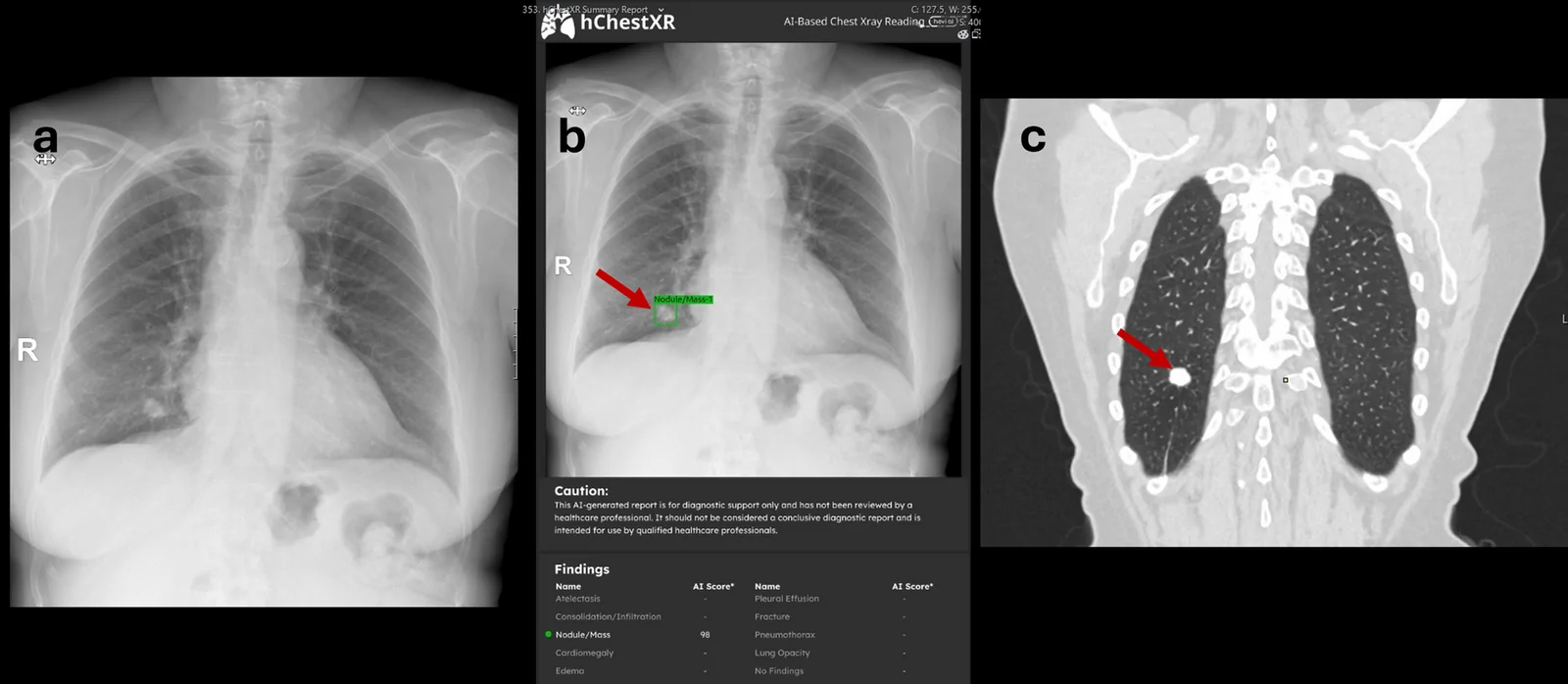
A 69-year-old female patient underwent a chest x-ray exam for a routine check-up in center A. (**a**) Posteroanterior chest radiograph of the patient. (**b**) The AI system identified a nodule/mass in the right lower lung zone (arrow). (**c**) The corresponding coronal CT image demonstrates a nodule in the right lower lobe (arrow). This case was classified as a true positive for the AI system. The subsequent biopsy confirmed lung cancer.

**Fig. 4:**
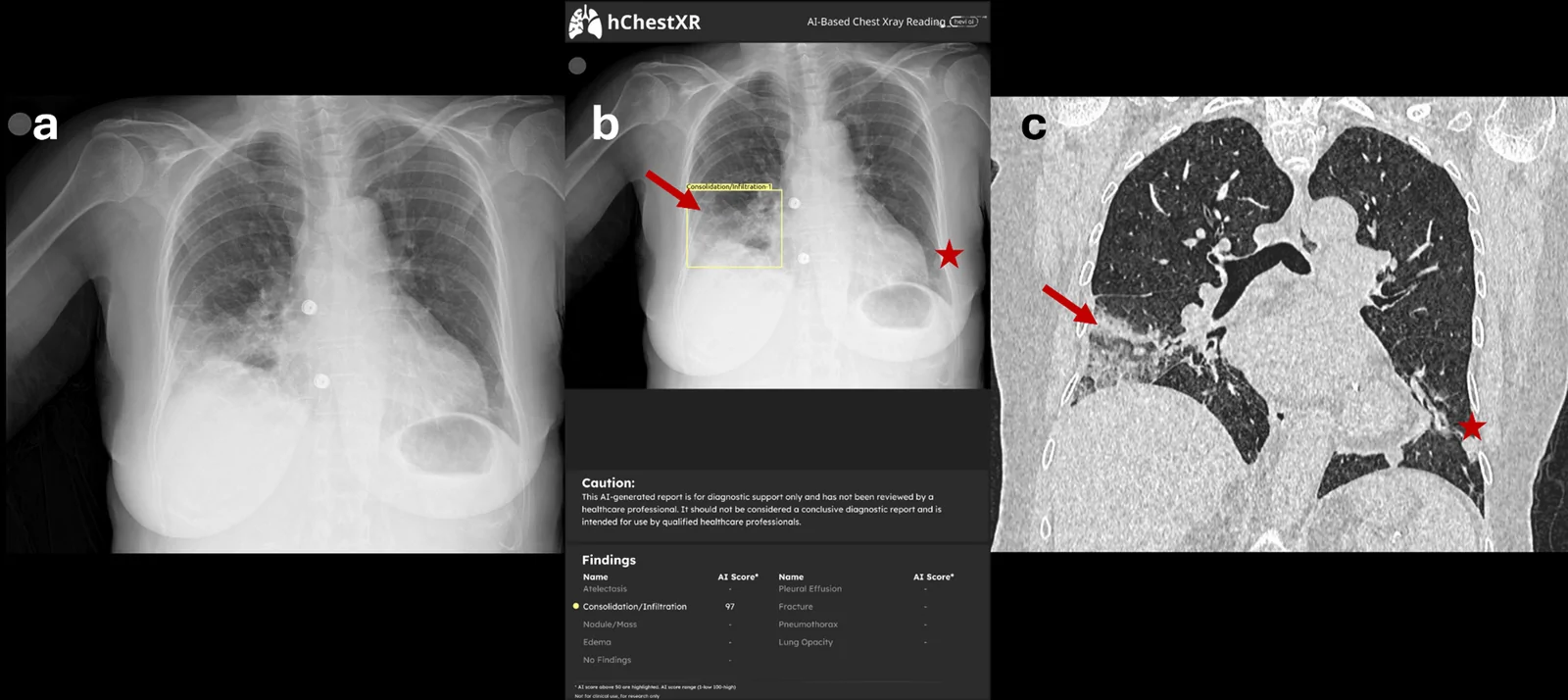
A 58-year-old female under surveillance for bladder cancer with a symptom of back and chest pain in Center C. (**a**) Posteroanterior chest radiograph of the patient. (**b**) The AI system identified the consolidation (arrow) in the right lower lung zone, yet it missed the visible nodule/mass in the left lower zone (star). (**c**) The corresponding coronal CT image demonstrates the consolidation in the right middle lobe (arrow) and solid subpleural lung nodule in the left lower lobe (star) missed by the AI system. This case was classified as true positive for the consolidation and false negative for the nodule.

**Fig. 5:**
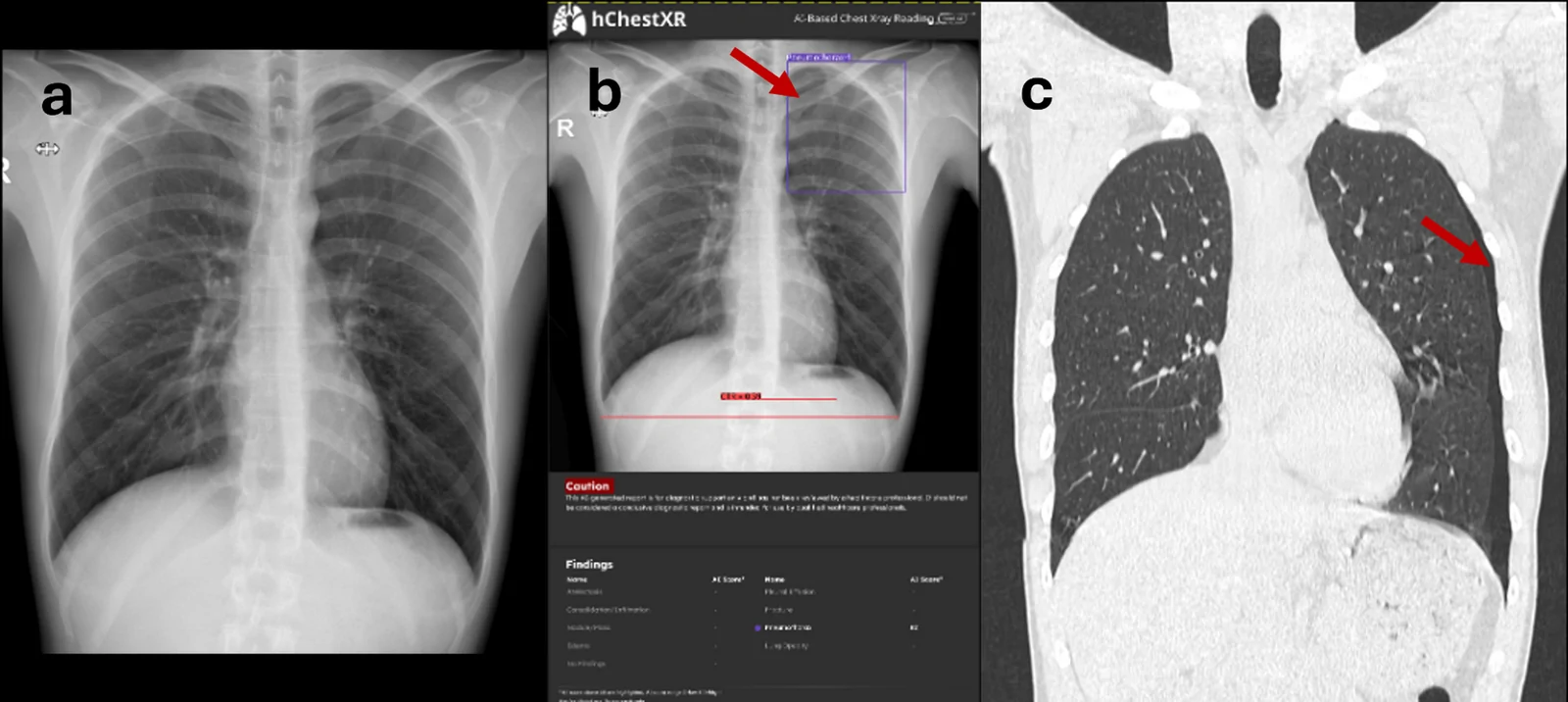
A 30-year-old male patient presented to the emergency department with chest pain in Center D. (**a**) Posteroanterior chest radiograph of the patient. (**b**) The AI system identified the pneumothorax in the left upper zone (arrow). (**c**) The corresponding coronal CT image demonstrates the pneumothorax in the left hemithorax (arrow). This case was classified as true positive.

Using the CT-anchored radiographic-detectability reference standard, 698 radiographically visible CT-confirmed target abnormalities were identified in 441 patients. The most common reference-positive findings were pleural effusion, present in 197 of 940 examinations (21.0%); consolidation/opacity, present in 172 of 940 examinations (18.3%); and atelectasis, present in 143 of 940 examinations (15.2%). Less prevalent findings included nodule/mass in 91 examinations (9.7%), pulmonary edema in 42 examinations (4.5%), fracture in 32 examinations (3.4%), and pneumothorax in 21 examinations (2.2%). Predefined CXR-inapparent CT findings, including nodules/masses smaller than 6 mm, subsegmental atelectasis, and trace pleural effusions, were excluded from the primary radiographic-detectability analysis. Representative AI outputs are shown in Figs. 3, 4 and 5.

Overall diagnostic performance at the manufacturer’s default threshold is summarized in Table 1. Sensitivity was highest for fracture (91%; 95% CI: 76–97) and pneumothorax (90%; 95% CI: 71–97), followed by pleural effusion (84%; 95% CI: 79–89), nodule/mass (77%; 95% CI: 67–84), consolidation/opacity (77%; 95% CI: 71–83), pulmonary edema (74%; 95% CI: 59–85), and atelectasis (64%; 95% CI: 56–72). Specificity was highest for fracture and pneumothorax, both 98%, and ranged from 82% for consolidation/opacity to 95% for pulmonary edema among the remaining findings. Accuracy ranged from 81% for consolidation/opacity to 98% for pneumothorax; however, accuracy was interpreted as a secondary descriptive metric because abnormality prevalence varied substantially across findings.

**Table 1:** Overall default-threshold operating-point performance of the AI system.

Finding	Positive/*N* (%)	TP	FP	TN	FN	Sensitivity % (95% CI)	Specificity % (95% CI)	PPV % (95% CI)	NPV % (95% CI)	FP among AI+ %	FP/100 exams
Atelectasis	143/940 (15.2)	92	73	724	51	64 (56–72)	91 (89–93)	56 (48–63)	93 (91–95)	44.2	7.8
Nodule/mass	91/940 (9.7)	70	91	758	21	77 (67–84)	89 (87–91)	43 (36–51)	97 (96–98)	56.5	9.7
Consolidation/opacity	172/940 (18.3)	133	141	627	39	77 (71–83)	82 (79–84)	49 (43–54)	94 (92–96)	51.5	15.0
Pleural effusion	197/940 (21.0)	166	99	644	31	84 (79–89)	87 (84–89)	63 (57–68)	95 (94–97)	37.4	10.5
Pulmonary edema	42/940 (4.5)	31	41	857	11	74 (59–85)	95 (94–97)	43 (32–55)	99 (98–99)	56.9	4.4
Fracture	32/940 (3.4)	29	21	887	3	91 (76–97)	98 (96–98)	58 (44–71)	100 (99–100)	42.0	2.2
Pneumothorax	21/940 (2.2)	19	21	898	2	90 (71–97)	98 (97–99)	48 (33–63)	100 (99–100)	52.5	2.2

CI, confidence interval; FN, false negative; FP, false positive; NPV, negative predictive value; PPV, positive predictive value; TN, true negative; TP, true positive.

Predictive values were interpreted in relation to abnormality prevalence. NPV was high across all findings, ranging from 93% for atelectasis to 100% for fracture and pneumothorax. PPV was more modest, ranging from 43% for nodule/mass and pulmonary edema to 63% for pleural effusion. At the evaluated operating point, the proportion of AI-positive outputs that were false positive was 56.5% for nodule/mass, 56.9% for pulmonary edema, 52.5% for pneumothorax, 51.5% for consolidation/opacity, 44.2% for atelectasis, 42.0% for fracture, and 37.4% for pleural effusion. Expressed per 100 examinations, false-positive outputs were most frequent for consolidation/opacity (15.0 per 100 examinations), followed by pleural effusion (10.5), nodule/mass (9.7), atelectasis (7.8), pulmonary edema (4.4), fracture (2.2), and pneumothorax (2.2).

Center-level prevalence and operating-point performance varied across institutions, as summarized descriptively in Table 2 and Supplementary Tables S1a and S1b. These analyses were considered exploratory because several findings had sparse positive counts at individual centers, particularly pneumothorax, fracture, and pulmonary edema, and some center-specific strata contained very few or no positive cases. Therefore, center-level differences should be interpreted as descriptive evidence of performance variability across institutions rather than definitive evidence of site-level performance differences or specific domain-shift mechanisms.

**Table 2:** Center-level prevalence and performance variability.

Finding	Center-specific positive cases, *n*/*N* (%)	Sensitivity range %	Specificity range %	PPV range %	NPV range %	*p* for sensitivity	*p* for specificity	*p* for PPV
Atelectasis	A: 5/299 (1.7); B: 69/250 (27.6); C: 51/312 (16.3); D: 18/79 (22.8)	56–68	81–100	5–100	88–99	< 0.001	< 0.001	< 0.001
Nodule/mass	A: 12/299 (4.0); B: 47/250 (18.8); C: 24/312 (7.7); D: 8/79 (10.1)	58–83	83–94	13–69	95–99	< 0.001	< 0.001	< 0.001
Consolidation/opacity	A: 14/299 (4.7); B: 69/250 (27.6); C: 67/312 (21.5); D: 22/79 (27.8)	43–86	73–92	7–72	89–96	0.25	< 0.001	< 0.001
Pleural effusion	A: 13/299 (4.3); B: 76/250 (30.4); C: 82/312 (26.3); D: 26/79 (32.9)	69–91	81–95	14–89	86–98	< 0.001	< 0.001	< 0.001
Pulmonary edema	A: 1/299 (0.3); B: 15/250 (6.0); C: 26/312 (8.3); D: 0/79 (0.0)	67–100	90–97	0–69	98–100	< 0.001	< 0.001	< 0.001
Fracture	A: 1/299 (0.3); B: 13/250 (5.2); C: 12/312 (3.8); D: 6/79 (7.6)	85–100	97–99	10–86	99–100	< 0.001	0.001	0.001
Pneumothorax	A: 0/299 (0.0); B: 17/250 (6.8); C: 1/312 (0.3); D: 3/79 (3.8)	88–100	96–100	0–100	99–100	< 0.001	< 0.001	< 0.001

NPV, negative predictive value; PPV, positive predictive value.

Intended as an exploratory descriptive summary of center-level variability. Because several findings had sparse positive counts at individual centers, including pneumothorax, fracture, and pulmonary edema, center-level estimates should not be interpreted as definitive evidence of site-level performance differences or as proof of specific domain-shift mechanisms.

## Discussion

This retrospective bi-national multicenter study evaluated the locked default-threshold operating-point performance of a commercial chest radiograph AI system. The analysis focused on selected CT-confirmed thoracic findings adjudicated for radiographic visibility. The system performed best for high-conspicuity findings, particularly pneumothorax and fractures. In contrast, sensitivity and PPV were lower for more subtle or diffuse abnormalities. Center-level variability further supports the need for local validation and post-deployment monitoring before routine clinical implementation.

The diagnostic performance observed in our study is broadly consistent with prior multicenter validations of commercial chest radiograph AI systems. However, direct numerical comparisons should be interpreted cautiously because reference standards, cohort composition, and reported metrics differ across studies. Many published evaluations use radiologist interpretation of the index radiograph as the reference standard. In contrast, our study used temporally paired chest CT and evaluated CT-confirmed abnormalities adjudicated as radiographically visible on CXR. CT pairing was permitted up to 14 days for relatively stable findings. For potentially rapidly evolving abnormalities, including pneumothorax, pleural effusion, consolidation/opacity, and pulmonary edema, the allowable CXR–CT interval was restricted to ≤ 2 days.

There is precedent for CT-anchored evaluation of commercially available chest radiograph AI. Tejani et al. validated a commercial deep-learning system (INSIGHT CXR; Lunit) for pneumothorax and pleural effusion detection using CT obtained within 24 h as ground truth, reporting AUROC-based performance and workflow impact through reduced resident reading time and improved inter-reader concordance. In that pleural-pathology–enriched cohort, AI achieved AUROC values of approximately 0.80 for pneumothorax and 0.82 for pleural effusion, and AI assistance improved resident efficiency and agreement. This evaluation framework differs from our real-world multicenter cohort, in which we report thresholded operating-point metrics and observed high pneumothorax performance at the default threshold under a strict ≤ 2-day CT pairing window for dynamic entities^17^.

The hybrid CT-confirmed, CXR-visible reference framework should also be considered when interpreting apparent AI errors. Determination of radiographic visibility relied on reader consensus and was not supported by independent inter-reader agreement statistics. Therefore, some apparent false-positive or false-negative AI outputs may partly reflect uncertainty in the reference standard rather than unequivocal model error, particularly for borderline or subtle findings such as mild atelectasis, small nodules/masses, early pulmonary edema, and equivocal consolidation/opacity.

Similarly, Kim et al. evaluated a commercial algorithm (Insight CXR; Lunit) in a multicenter health screening cohort using CT-derived ground truth and demonstrated that apparent performance depends materially on how “ground truth” is defined with respect to CXR detectability. Specifically, they reported lower performance when using CT-only ground truth and higher performance when restricting positives to CT-confirmed abnormalities that were also called on CXR, supporting the methodological rationale for our visibility-adjudicated evaluation of CT-confirmed findings against CXR detectability limits^14^.

Within this framework, pneumothorax performance remained high at the evaluated default threshold. The magnitude of performance is broadly comparable to multicenter reports for FDA-cleared and CE-marked commercial tools, including Annalise Enterprise CXR and Lunit INSIGHT CXR. However, direct numerical comparison is limited because those studies often reported AUROC or used CXR-based reference standards^19,20^. The lower PPV in our cohort is likely related to low pneumothorax prevalence, consistent with the prevalence dependence of PPV^5^. More broadly, CT-informed reference standards can reduce label noise compared with radiograph-only labeling. However, they may also increase apparent task difficulty by identifying CT-confirmed abnormalities with only subtle or borderline radiographic manifestations. This was one reason for including visibility adjudication in our study design.

For fracture detection, our results fall within the range reported for other commercial algorithms evaluated in trauma-enriched or emergency-care cohorts^7^. Studies relying on radiograph-based labeling may undercount subtle fractures that are occult or equivocal on CXR, whereas a CT-informed reference standard may improve lesion ascertainment while also accentuating difficulty when only faint correlates are present on the radiograph. Consistent with clinical experience, performance is generally expected to be higher for displaced or cortical fractures and lower for subtle rib or clavicular injuries, which remain challenging for both AI systems and human readers.

For pleural effusion and consolidation/opacity, performance was broadly comparable to large-scale evaluations using CXR-based reference standards^6,15^. In contrast, atelectasis and pulmonary edema showed more modest sensitivity, consistent with the subtle, diffuse, and low-contrast radiographic appearance of these entities^20^. Here, reference standard definitions are particularly consequential: radiograph-only ground truth may miss early or mild disease, whereas CT-based adjudication can identify disease more reliably but may also include findings with limited radiographic conspicuity.

For nodules/masses, the AI system demonstrated moderate sensitivity and specificity, consistent with the known limitations of CXR for small or low-contrast lesions. Prior studies using different reference-standard paradigms have reported a wide range of nodule performance depending on whether the reference standard was radiograph-based, CT-adjudicated, or hybrid^5^. When CT informs ground truth, apparent task difficulty increases because CT reveals lesions that may be borderline or only weakly manifested on radiographs. Accordingly, our CT-paired, visibility-adjudicated design provides a conservative estimate of performance for radiographic detection of CT-confirmed nodules/masses.

Diagnostic performance varied across centers, consistent with the practical challenge of transporting chest radiograph AI systems across institutions. This heterogeneity may reflect differences in case mix, abnormality prevalence, PA versus AP projection, portable versus fixed-unit acquisition, detector/vendor characteristics, image post-processing, CT utilization patterns, and local clinical workflow. Although some center-level differences were statistically significant, the retrospective design of this study does not allow causal attribution to specific technical or clinical factors. Therefore, these findings should be interpreted as evidence of performance variability rather than proof of a particular domain-shift mechanism. Collectively, they support the need for local validation, site-level performance monitoring, and post-deployment surveillance even for AI systems trained on heterogeneous multicenter datasets.

Although the AI system maintained high NPV across evaluated abnormalities, this should not be interpreted as evidence that the system can safely exclude disease in isolation. NPV is strongly prevalence-dependent and increases when abnormality prevalence is low. Therefore, the high NPV observed in this CT-paired cohort should be interpreted alongside sensitivity, disease prevalence, clinical context, false-positive burden, and local performance monitoring. The findings may support further evaluation as radiologist-supervised decision support. However, this study did not include a human-reader comparison, AI-assisted reader experiment, reading-time analysis, report-turnaround evaluation, or patient-outcome assessment. Therefore, it cannot establish radiologist equivalence, incremental reader benefit, triage effectiveness, workflow improvement, or clinical outcome impact. These endpoints require prospective reader and implementation studies.

The modest PPV observed for several findings is clinically important. At the evaluated default threshold, many AI-positive outputs represented false positives. In clinical deployment, false-positive outputs may increase radiologist workload, reduce user trust, and contribute to alert fatigue, especially if presented as interruptive alerts. Therefore, specificity, PPV, and false-positive burden should be considered alongside sensitivity when evaluating suitability for a given clinical workflow. Whether such systems improve preliminary screening, prioritization, or reporting efficiency should be tested in prospective implementation studies^21,22^.

Several limitations should be emphasized. First, because inclusion required both CXR and clinically indicated chest CT, the cohort was CT-verified and likely pathology-enriched, introducing verification and selection bias. Therefore, prevalence-dependent metrics such as PPV and NPV should not be extrapolated to routine all-comer CXR populations with different abnormality prevalence. Second, the CT-confirmed, CXR-visible reference standard was hybrid and partly subjective. Although this approach was intended to align CT-confirmed abnormalities with radiographic detectability, radiographic visibility was determined by consensus without independent inter-reader agreement analysis, and some apparent AI errors may reflect reference-standard uncertainty. Third, continuous probability scores were not available; therefore, AUROC, calibration, Brier score, threshold-sensitivity, and decision-curve analyses could not be performed. Fourth, the study did not include a human-reader comparison, workflow assessment, reporting-time analysis, or patient-outcome evaluation. Fifth, temporal mismatch between CT and CXR may have introduced misclassification despite the restricted interval for rapidly evolving findings. Sixty, center-level analyses were exploratory and underpowered for rare findings, and the study was not designed to identify causal mechanisms for site-level variability. Finally, AI system was evaluated as a predefined-label detection and localization tool rather than as a comprehensive chest radiograph interpretation system. Although the software provides class-specific bounding boxes and predefined textual output in a PACS-viewable secondary-capture/PDF report, this study did not evaluate free-text report generation, report completeness, lesion-size estimation, or formal lesion counting. In addition, localization was assessed only for determining concordance between AI-positive outputs and reference abnormalities; quantitative box-level localization performance and lesion-level sensitivity for every individual abnormality were not evaluated. In examinations with multiple lesions of the same class, detection of at least one location-concordant target lesion was sufficient for examination-level true-positive classification.

In summary, this bi-national multicenter study provides CT-anchored validation of a CE-certified AI system for chest radiograph interpretation using a detectability-aligned reference framework. The system demonstrated strong default-threshold performance for pneumothorax and fractures, with more modest sensitivity for subtle entities such as atelectasis and pulmonary edema, and center-level variability highlighting possible transportability challenges. These findings support further prospective evaluation of the system as radiologist-supervised decision support, particularly in settings where local validation and post-deployment monitoring can be maintained. Future work should include prospective reader and implementation studies to quantify workflow and clinical endpoints, including reading time, report turnaround, and downstream decisions, and to characterize performance across diverse acquisition settings and patient populations.

## Supplementary Information

Below is the link to the electronic supplementary material.

Supplementary Material 1.

## Data Availability

The datasets generated during and/or analyzed during the current study are available from the corresponding author on reasonable request.
