# Supplementary material for: Default-threshold operating-point validation of a commercial chest radiograph AI system for selected CT-anchored thoracic findings: a bi-national multicenter retrospective study
Source: Sci Rep. 2026 Jul 25;16:26052. doi: 10.1038/s41598-026-55487-9 (PMC13490484; doi:10.1038/s41598-026-55487-9)
Supplement: Supplementary file 1 [file 41598_2026_55487_MOESM1_ESM.docx]

**Supplementary Table S1a. Full center-specific confusion matrices and prevalence.**

| Finding | Center | N | Positive/N (%) | TP | FP | TN | FN | FP/100 exams |
| --- | --- | --- | --- | --- | --- | --- | --- | --- |
| Atelectasis | A | 299 | 5/299 (1.7) | 3 | 57 | 237 | 2 | 19.1 |
| Atelectasis | B | 250 | 69/250 (27.6) | 47 | 8 | 173 | 22 | 3.2 |
| Atelectasis | C | 312 | 51/312 (16.3) | 32 | 8 | 253 | 19 | 2.6 |
| Atelectasis | D | 79 | 18/79 (22.8) | 10 | 0 | 61 | 8 | 0.0 |
| Nodule | A | 299 | 12/299 (4.0) | 7 | 46 | 241 | 5 | 15.4 |
| Nodule | B | 250 | 47/250 (18.8) | 37 | 17 | 186 | 10 | 6.8 |
| Nodule/mass | C | 312 | 24/312 (7.7) | 20 | 16 | 272 | 4 | 5.1 |
| Nodule/mass | D | 79 | 8/79 (10.1) | 6 | 12 | 59 | 2 | 15.4 |
| Consolidation/opacity | A | 299 | 14/299 (4.7) | 6 | 78 | 207 | 8 | 26.1 |
| Consolidation/opacity | B | 250 | 69/250 (27.6) | 59 | 28 | 153 | 10 | 11.2 |
| Consolidation/opacity | C | 312 | 67/312 (21.5) | 51 | 20 | 225 | 16 | 6.4 |
| Consolidation/opacity | D | 79 | 22/79 (27.8) | 17 | 15 | 42 | 5 | 19.0 |
| Pleural effusion | A | 299 | 13/299 (4.3) | 9 | 55 | 231 | 4 | 18.4 |
| Pleural effusion | B | 250 | 76/250 (30.4) | 63 | 8 | 166 | 13 | 3.2 |
| Pleural effusion | C | 312 | 82/312 (26.3) | 75 | 26 | 204 | 7 | 8.3 |
| Pleural effusion | D | 79 | 26/79 (32.9) | 19 | 10 | 43 | 7 | 12.7 |
| Pulmonary edema | A | 299 | 1/299 (0.3) | 1 | 17 | 281 | 0 | 5.7 |
| Pulmonary edema | B | 250 | 15/250 (6.0) | 10 | 7 | 228 | 5 | 2.8 |
| Pulmonary edema | C | 312 | 26/312 (8.3) | 20 | 9 | 277 | 6 | 2.9 |
| Pulmonary edema | D | 79 | 0/79 (0.0) | 0 | 8 | 71 | 0 | 10.1 |
| Fracture | A | 299 | 1/299 (0.3) | 1 | 9 | 289 | 0 | 3.0 |
| Fracture | B | 250 | 13/250 (5.2) | 11 | 4 | 233 | 2 | 1.6 |
| Fracture | C | 312 | 12/312 (3.8) | 11 | 7 | 293 | 1 | 2.2 |
| Fracture | D | 79 | 6/79 (7.6) | 6 | 1 | 72 | 0 | 1.4 |
| Pneumothorax | A | 299 | 0/299 (0.0) | 0 | 12 | 287 | 0 | 4.0 |
| Pneumothorax | B | 250 | 17/250 (6.8) | 15 | 2 | 231 | 2 | 0.8 |
| Pneumothorax | C | 312 | 1/312 (0.3) | 1 | 7 | 304 | 0 | 2.2 |
| Pneumothorax | D | 79 | 3/79 (3.8) | 3 | 0 | 76 | 0 | 0.0 |

**Supplementary Table S1b. Full center-specific operating-point performance metrics.**

| Finding | Center | Accuracy % (95% CI) | Sensitivity % (95% CI) | Specificity % (95% CI) | PPV % (95% CI) | NPV % (95% CI) |
| --- | --- | --- | --- | --- | --- | --- |
| Atelectasis | A | 80 (75–84) | 60 (23–88) | 81 (76–85) | 5 (2–14) | 99 (97–100) |
| Atelectasis | B | 88 (83–91) | 68 (56–78) | 96 (92–98) | 85 (74–92) | 89 (84–92) |
| Atelectasis | C | 91 (88–94) | 63 (49–75) | 97 (94–98) | 80 (65–90) | 93 (89–95) |
| Atelectasis | D | 90 (81–95) | 56 (34–75) | 100 (94–100) | 100 (72–100) | 88 (79–94) |
| Nodule/mass | A | 83 (78–87) | 58 (32–81) | 84 (79–88) | 13 (7–25) | 98 (95–99) |
| Nodule/mass | B | 89 (85–92) | 79 (65–88) | 92 (87–95) | 69 (55–79) | 95 (91–97) |
| Nodule/mass | C | 94 (90–96) | 83 (64–93) | 94 (91–97) | 56 (40–70) | 99 (96–99) |
| Nodule/mass | D | 82 (72–89) | 75 (41–93) | 83 (73–90) | 33 (16–56) | 97 (89–99) |
| Consolidation/opacity | A | 71 (66–76) | 43 (21–67) | 73 (67–77) | 7 (3–15) | 96 (93–98) |
| Consolidation/opacity | B | 85 (80–89) | 86 (75–92) | 85 (79–89) | 68 (57–77) | 94 (89–97) |
| Consolidation/opacity | C | 88 (84–92) | 76 (65–85) | 92 (88–95) | 72 (60–81) | 93 (89–96) |
| Consolidation/opacity | D | 75 (64–83) | 77 (57–90) | 74 (61–83) | 53 (36–69) | 89 (77–95) |
| Pleural effusion | A | 80 (75–84) | 69 (42–87) | 81 (76–85) | 14 (8–25) | 98 (96–99) |
| Pleural effusion | B | 92 (88–94) | 83 (73–90) | 95 (91–98) | 89 (79–94) | 93 (88–96) |
| Pleural effusion | C | 89 (86–92) | 91 (83–96) | 89 (84–92) | 74 (65–82) | 97 (93–98) |
| Pleural effusion | D | 78 (68–86) | 73 (54–86) | 81 (69–89) | 66 (47–80) | 86 (74–93) |
| Pulmonary edema | A | 94 (91–96) | 100 (21–100) | 94 (91–96) | 6 (1–26) | 100 (99–100) |
| Pulmonary edema | B | 95 (92–97) | 67 (42–85) | 97 (94–99) | 59 (36–78) | 98 (95–99) |
| Pulmonary edema | C | 95 (92–97) | 77 (58–89) | 97 (94–98) | 69 (51–83) | 98 (95–99) |
| Pulmonary edema | D | 90 (81–95) | NE | 90 (81–95) | 0 (0–32) | 100 (95–100) |
| Fracture | A | 97 (94–98) | 100 (21–100) | 97 (94–98) | 10 (2–40) | 100 (99–100) |
| Fracture | B | 98 (95–99) | 85 (58–96) | 98 (96–99) | 73 (48–89) | 99 (97–100) |
| Fracture | C | 97 (95–99) | 92 (65–99) | 98 (95–99) | 61 (39–80) | 100 (98–100) |
| Fracture | D | 99 (93–100) | 100 (61–100) | 99 (93–100) | 86 (49–97) | 100 (95–100) |
| Pneumothorax | A | 96 (93–98) | NE | 96 (93–98) | 0 (0–24) | 100 (99–100) |
| Pneumothorax | B | 98 (96–99) | 88 (66–97) | 99 (97–100) | 88 (66–97) | 99 (97–100) |
| Pneumothorax | C | 98 (95–99) | 100 (21–100) | 98 (95–99) | 12 (2–47) | 100 (99–100) |
| Pneumothorax | D | 100 (95–100) | 100 (44–100) | 100 (95–100) | 100 (44–100) | 100 (95–100) |

*NE: Not estimated
